# Minding our Minds: Obsessive-Compulsiveness, Psychiatry, and Psychology

**DOI:** 10.1007/s11013-022-09767-4

**Published:** 2022-01-22

**Authors:** Lawrence D. Blum

**Affiliations:** 2400 Chestnut St., Suite 2810, Philadelphia, PA 19103 USA

**Keywords:** Obsessive–compulsive, Cognitive-Behavioral Therapy (CBT), Diagnostic and Statistical Manual (DSM), Defense mechanisms

## Abstract

Obsessive–compulsive features are commonly found in high-achieving people including psychiatrists, psychologists, and scientists. These traits have a substantial but unrecognized cultural influence on psychiatric and psychological science and practice. This article reviews obsessive–compulsive mechanisms and discusses the ways they both promote and impede psychiatric and psychological science and practice. It examines them in relation to two of the dominant psychiatric and psychological paradigms of our era, the Diagnostic and Statistical Manual (DSM), and Cognitive-Behavioral Therapy. Finally, the article suggests that better awareness of our collective obsessive–compulsive tendencies can facilitate a cultural shift toward a broader, more useful science of mind and brain, as well as therapies informed by more comprehensive scientific understanding.

## Introduction

There are striking parallels between standard scientific methods and the psychological phenomena that characterize obsessive–compulsive individuals. The shared characteristics include an orientation toward material reality, interest in rules and abstract thinking, attention to detail, conscientiousness, and a tendency to diminish the role of emotion. In both science and with individuals, these features can have productive and counterproductive, adaptive and maladaptive qualities. In this essay I discuss how obsessive–compulsive tendencies have promoted psychiatric and psychological science, as well as how excesses of them have impeded science, particularly by valuing procedure at the expense of meaning, the quantitative over the qualitative, and cognition over emotion. I note some evidence that psychiatric and psychological culture appears to be shifting to diminish some of these excesses, and I argue that increased awareness of obsessive–compulsive mechanisms can facilitate improved psychiatric and psychological science that is more comprehensive and more inclusive of the mind. I will first review obsessive–compulsive mechanisms, then discuss them in relation to two of the dominant psychiatric and psychological paradigms of our era, the Diagnostic and Statistical Manual (DSM), and Cognitive-Behavioral Therapy (CBT), and finally draw a few broader conclusions.

While the term obsessive–compulsive may bring to mind certain symptoms, such as repetitive thoughts or actions, what is important in comparing scientific methods and obsessive–compulsive phenomena is not a focus on symptoms but on a characteristic set of defense mechanisms. Defense mechanisms are (usually) unconscious mental operations that help people to manage their emotions and adjust to reality; as mentioned, they can be both adaptive and maladaptive (Vaillant et al. 1986). On the helpful side, these mental mechanisms protect people from uncomfortable or painful emotions, such as feeling sad, envious, ashamed, guilty, lustful, greedy, angry, etc. They also serve to keep intolerable wishes and fantasies out of mind and to limit the deleterious effects these uncomfortable or intolerable feelings and fantasies might have on the individual. In addition, they sometimes facilitate turning these feelings, wishes, and fantasies in a constructive direction. On the other hand, these defenses themselves can be limiting, and they are often needless holdovers from earlier periods of development, restrictions that individuals have not yet realized are no longer necessary to keep them safe.

The principal obsessive–compulsive defense mechanisms include isolation of affect, reaction formation, intellectualization, compartmentalization, rigidity of thought and behavior, the combination of doing and undoing, and doubting (Shapiro 1965; Gabbard 1994). They are typically accompanied by exaggerated feelings of responsibility and conscientiousness, as noted first by psychoanalytic observers (Freud 1908b; Paul 1996; Shapiro 1965) and then incorporated in the DSM (American Psychiatric Association 1980). Recent neuroscientific studies of obsessive–compulsive patients have described mental mechanisms clearly related to those mentioned above, but using different terminology, including conflict and error monitoring, task switching and reversal, response inhibition, perseverative cognition, and problems in decision-making and reward processing (Stern and Taylor 2014). A study of patients with obsessive–compulsive disorder (OCD) conducted from a cognitive-behavioral viewpoint provided empirical confirmation of magnified “responsibility beliefs” in these patients (Salkovskis et al. 2000).

Despite the advent of terminology from additional perspectives, the traditional, psychoanalytically derived terms for the obsessive–compulsive defense mechanisms continue to have primary usage across a broad range of therapies, and I will discuss them here in relation to their consequences for science and psychotherapy. *Isolation of affect* can facilitate extended periods of concentration and work, but it also contributes to a diminishment of emotional experience; in doing so it deprives significant aspects of mental life of their meaning (Freud 1909). *Reaction formation* is a common mechanism by which obsessive individuals try to deal with hostility by transforming negative feelings to positive ones; it may contribute to the efforts of scientists to contribute to the public good, as well as transforming anger into constructive intellectual debate. It can also make it difficult for therapists to tune into patients’ anger and destructiveness. *Intellectualization* has obvious benefits for the pursuit of science, but can discourage the use of intuition and diminish emotional sensitivity. Closely related to intellectualization, *compartmentalization*, the inclination to put things in separate categories, promotes the tendency to classify and quantify, common obsessive–compulsive features that have obvious utility for science—all of our essential taxonomies of animals, plants, and diseases are useful examples of benefit from this disposition. On the other hand, too much compartmentalization denies, even severs, meaningful connections. *Rigidity of thought and behavior* can promote a useful single-minded focus and determination, but it can also compromise creativity and exploration. Likewise, a degree of *doubt* and need for proof are beneficial, but too much doubt can be incapacitating. It is worth adding that the familiar, emotionless obligatory repetitions of obsessive thoughts or compulsive actions can also facilitate intense concentration and work, but, like isolation of affect and compartmentalization, tend to diminish meaning.

Additional typical obsessive–compulsive features described by Freud include *orderliness*, *obstinacy*, and *penuriousness* (Freud 1908a); these have been incorporated into the DSM in connection with obsessive–compulsive personality disorder. Orderliness and a degree of obstinacy, the latter of which especially aids persistence in the face of difficulty, have obvious benefits for scientific pursuits, but can lead therapists to impose their own obsessive structures on patients’ narratives. As a group, the characteristic obsessive–compulsive defense mechanisms tend to promote an orientation to material reality, as opposed to daydreaming and fantasy, a preoccupation with time (and thus inclinations toward punctuality or procrastination), and a sense of moral conscientiousness.

Some of the compelling advantages and disadvantages of obsessive–compulsive defense mechanisms for medical practice were described by Gabbard (1985) in an article titled “The Role of Compulsiveness in the Normal Physician.” It is no coincidence that most doctors have obsessive–compulsive features to one extent or another. The isolation of affect allows doctors to see dying patients and still retain enough emotional distance to go about the rest of their work, but it also may make it difficult for them to fully tune in to their patients’ emotional struggles. Likewise the need for repetitive checking can diminish the frequency of errors, but can also prevent progress and impede decision-making. The utility of an obsessive–compulsive disposition for the practice of medicine applies equally well to many areas of science. Individuals with an obsessive–compulsive tendency may thus be inclined to select medical and scientific careers, and may thrive in them, creating a significant, although little-noticed, obsessive–compulsive quality to medical and scientific culture.

As noted above, the mechanisms of isolation of affect, compartmentalization, doubting, and repetition, and their use to keep difficult feelings and ideas at bay lend themselves to scientific pursuits involving measurement, procedure, and classification better than to areas of science more closely related to emotion, intuition, or subjective experience. These dichotomous considerations hold for science generally, but have particular implications for psychology, psychiatry, and the practice of psychotherapy. In psychoanalysis, for example, the use of the couch is intended in part to allow access to more emotional experience, but it can also be used by either patient or analyst to isolate affect and keep an emotional distance. Although psychoanalysis specifically attends to emotion, and certainly has some flexibility of thought and practice, it has also at times suffered from rigid thought and orthodoxy that has had an obsessive character. The degree to which psychoanalytic treatments should rely on insight, which necessarily has a cognitive component, versus immersion in new forms of emotional and relational experience, remains an active area of debate within the field. Psychoanalysis, has not, however, produced the sort of compartmentalized classification system of the DSM or the repetitive, cognition-oriented techniques of CBT, which appear to be built on highly ordered structures. The scientific culture in which the DSM and CBT are so prominent, however, may gradually be changing. Psychiatrists and psychologists are beginning to acknowledge the limitations of the symptom checklist procedures of the American Psychiatric Association’s DSM (Galatzer-Levy and Galatzer-Levy 2007), and they have begun to pay more attention to emotions, in areas in which they have been downplayed, such as CBT (Thoma and McKay 2014; Roy-Byrne 2017) and cognitive neuroscience (Panksepp et al. 2017). The DSM and CBT provide useful examples of the advantages and disadvantages of obsessive-compulsiveness in psychiatric and psychological science, as well as illustrations of gradual cultural changes.

## The DSM

After 6 years in development, the DSM III (American Psychiatric Association 1980) was published 1980 in an effort to rationalize psychiatric diagnosis and facilitate research, to remove it from a foundation in psychoanalytic principles present in DSM I and DSM II, and to fashion it more in accord with a medical model (Galatzer-Levy and Galatzer-Levy 2007). The move toward a medical model has been described as motivated in part to ward off competition from non-psychiatrists performing psychotherapy (Kawa and Giordano 2012). The authors of the DSM III claimed it was non-theoretical and suggested that such a thing was possible. The DSM III employed a positivist rationalism, attempting to base diagnoses only on what could be objectively observed. That it might be short-sighted in diagnosing humans to look only at objectively observable data (including patients’ self-reports), and not pay significant attention also to such important matters as feelings or fantasies, was not considered. The changed orientation of the DSM thus steered those using it toward psychotherapies focused on groups of symptoms rather than the whole person. The DSM III did away with the category of “neurosis” and separated the diagnostic labels into multiple new categories. “Phobic Neurosis” became five types of “Phobic Disorders,” and “Depressive Neurosis” became four categories of “Major Depression.”(American Psychiatric Association 1980; Kawa and Giordano 2012) The 182 diagnostic categories in DSM II expanded to 265 in DSM III, and then 297 in DSM IV (American Psychiatric Association 1994). The expanding number of discrete diagnostic categories also contributed to the DSMs’ ambition to parallel medical diagnosis, and this may have facilitated its prompt and broad acceptance not only because of its likeness to medical culture, but also because of our broad cultural familiarity and comfort with obsessive–compulsive tendencies.

Whatever their merits and demerits as diagnostic compendiums, these DSMs have the features of an expanding obsessive–compulsive construction. They show an increasing trend toward compartmentalization, with more and more categories and sub-categories, as if the peas and the potatoes on the plate should never overlap or touch each other. The isolation of affect is evident in the emphasis on what is objectively observable (Some emotional reactions are directly observable; others, such as imagining one is unattractive to ward off anxiety arising from romantic attention, are not). The counting of numbers of symptoms to formally make a diagnosis reflects orderliness, and again an attempt at objectivity, but attention to feelings, fantasy, and meaning are diminished. The document thus has all the advantages and disadvantages of an obsessive–compulsive approach to life. It has indeed facilitated empirical research, and perhaps especially research involving more severe disorders, but much DSM-inspired research has also been based on the idea of discrete clinical syndromes that rarely occur in life.

While in my experience most psychiatrists seem to have preferred to treat the DSMs as if they describe a proven reality not to be questioned, one might think it obvious that the DSMs, like all documents, are cultural and historical artifacts. Gaines’ (1992) essay on the ethnocentric construction of the DSMs describes the move toward an increased conceptual separation of an idealized autonomous self and an alien other (the patient), as well as increased division between mind and body, and a move toward the assumption of biological etiology. The trends Gaines observes are consistent with the obsessive–compulsive compartmentalization and de-emphasis of emotional experience and social engagement that are discussed here. Like any complex cultural phenomenon, the evolution of the DSMs has been multifactorial, and in addition to the factors noted above there were convergent influences on psychiatry by the insurance and pharmaceutical industries. The idea of separate, isolated diagnostic categories, and limited biologically based treatments appealed to insurers aiming to diminish their costs (Mayes and Horwitz 2005) and enabled drug companies to apply to the Food and Drug Administration for approval of drugs as officially indicated for newly constructed, supposedly discrete, “disease” constructs such as “social phobia.”

More recently, DSM 5 (American Psychiatric Association 2013), like DSM IV, furthers the obsessive trend of the multiplication of the diagnostic sub-categories, but also shows signs of tempering the obsessive categorization and counting of symptoms as it introduces a more dimensional approach to diagnosis and pays more attention to cultural influences in psychiatric syndromes (although it never questions itself as a representative of the preeminent culture). The recent increased interest in common factors in mental illnesses (Barch 2020), instead of regarding them all as discrete entities, may also be part of a diminishment of obsessive compartmentalization. Clinically we often see an individual’s obsessive–compulsive symptoms abate over time as the emotional pressures that underlie them diminish and the obsessive defenses are less needed. Perhaps we observe the equivalent of this now professionally, with the more extreme obsessiveness of the DSMs diminishing in the latest version as psychiatry’s anxieties of not being sufficiently medical, or of being too psychoanalytic, begin to decrease.

## Cognitive-Behavioral Therapy (CBT)

Cognitive behavioral therapy, perhaps even more than the DSM, has been built on, and has recently gradually begun to depart from (Thoma and McKay 2014; Roy-Byrne 2017), an obsessive–compulsive platform. In the 1950s Paul MacLean (1952) described what he termed the “limbic system” and pointed out that in evolution this neurobiological center of the emotions long preceded the cortical development that appears to support advanced cognition, an observation rapidly and widely accepted in evolutionary biology and emphatically confirmed by more recent neuroscience (Solms 2021). In the 1960s, however, Aaron Beck, the developer of Cognitive Therapy (which soon became CBT), insisted on the priority of cognition over emotion. Beck describes patients’ negative thoughts leading to uncomfortable feelings, and dismisses prior ideas that negative thoughts might result from emotional conflict or distress (Beck 1963). The psychoanalytic approach of the time likewise regarded patients’ negative thoughts as important, especially early in the treatment of depressed patients, but understood these thoughts as efforts to deal with underlying difficulties with loss, anger, and guilt (Mendelson 1974; Bird 1973). The cultural background of American pragmatism may have influenced CBT’s practical, procedure-oriented, and directive qualities, and surely provided fertile ground for CBT’s rapid expansion. CBT’s very name, which includes cognition and behavior and excludes emotion, underscores the obsessive isolation of affect in its origin.

Obsessive–compulsive mechanisms are featured in CBT’s therapeutic approach as well as in its theory. Repetitive exercises such as daily homework are prescribed, sometimes asking patients to quantify their distress with numerical ratings. Some of the exercises aim to undo negative thoughts by replacing them with realistic ones; in practice this is often replacing the negative with a positive, seeming to promote the obsessive–compulsive mechanism of reaction formation.

There is little if any evidence in most empirically studied treatments to demonstrate that specific putative therapeutic mechanisms, such as thought substitution efforts in CBT, contribute significantly to patients’ recoveries (Cuijpers et al. 2019). However, there is a good deal of evidence that the relationship with the patient, which has been downplayed in CBT, may have a significant influence on therapeutic outcome (Zilcha-Mano 2017; Horvath et al. 2011). In the majority of empirical studies of psychotherapies, the *meaning* of the interactions between therapist and patient has received little attention, and this is the case with CBT. In traditional CBT, the therapist takes a highly directive, authoritative role. For some patients, this may be reassuring; for others the implicit demand for submission breeds rebellion. In fact, a recent study of lying in psychotherapy found that among the most commonly lied about therapy-related topics were "pretending to do homework or take other actions suggested by my therapist," "pretending to like my therapist's comments or suggestions," and "my real opinion of the therapist" (Blanchard and Farber 2016). Such lies are likely to be more common when the therapist takes the role of unquestioned authority, and the relationship between patient and therapist is not part of the therapeutic discussion. Obsessive attention to procedure over meaning and relationship may thus compromise therapeutic effects. A similar preference for procedure over meaning has contributed to numerous claims of therapeutic success based on small statistical findings with no meaningful benefits to patients (Shedler 2018).

The matter of meaning, so essential to humans, is routinely overlooked in procedurally oriented therapies, and in studies of them. It is however, inescapable. For example, the prescription of homework exercises inevitably has particular meanings for each individual patient. Many depressed patients (as well as other patients, and people in general) long to be taken care of and the provision of exercises sometimes makes them feel that they have been tangibly given something, just as many patients visiting a primary care doctor want to leave with a prescription. Thus, if patients benefit from the exercises, we need to ask, is it from doing them, or from receiving the gift of them? Further, given how ubiquitous obsessive–compulsive defenses are in our society, and how many useful emotional purposes they serve, it is not surprising that CBT’s prescription of repetitive tasks that distract from uncomfortable feelings should benefit some people. The exercises may function as better obsessive–compulsive defenses, sometimes successfully replacing self-critical ruminations. It is also expectable, given the obsessive–compulsive trends of our society, that the procedural approach of CBT might be widely accepted by many, with the obsessive–compulsive qualities of it having a comfortable, almost invisible familiarity. For other patients, however, the exercises may be a burden which they must submit to or rebel against. As a further example, a typical CBT therapy manual advises patients, "Even if you are feeling down, see what it feels like to act 'as if' you feel good. Smile, even if you don't feel like smiling" (Miranda et al. 2008). Some patients may find this useful, but others have been known to react to such suggestions as indications that they will never be understood, which may increase despair. In all of these examples, it is the meaning that matters, and that is precisely what our scientific studies tend to overlook. The simple, clear regimens of CBT facilitate clinical practice and research of a sort, but of a limited sort. As with the more medically oriented DSMs, CBT achieved rapid and broad cultural acceptance, especially in places such as the United States with traditions of pragmatism and efficiency, and of valuing positivism and depreciating hermeneutics.

Just as with the DSM, CBT’s emphasis on the cognitive over the emotional, and the procedural over the experiential, has diminished in recent years. A compartmentalized approach with different CBTs for each of many different disorders has recently been challenged with the idea of “transdiagnostic” CBT (Barlow et al. 2017). Emotion is now more regularly taken into account to some degree, although the approach often continues to have a didactic quality with the therapist in the role of an unquestioned directive authority. An example of this is the recently popular Dialectical Behavior Therapy (DBT) for borderline personality disorder. Patients are taught to recognize and name emotions, as well as to delay putting them into action, all very important things for those who struggle with these matters (Linehan 2014). The incorporation of “mindfulness” has led to an increased emphasis on experiencing and tolerating emotions, whatever they may be. There is thus less obsessive avoidance of emotion, but the meaning of the emotions and how they relate to patients’ lives is not a major focus, nor is the relationship between therapist and patient examined or learned from. As with the DSM, in the CBT arena, we have an evolving culture, with a gradual diminution of the most rigid, restrictive obsessive–compulsive qualities. Nonetheless, these approaches do not take full account of the degree to which humans are intensely social animals with minds that work metaphorically to understand complex emotional and social processes.

## Discussion

Every psychologist and psychiatrist, every scientist and medical practitioner, every therapist, has to grapple with uncertainty on a daily basis. This inevitable uncertainty provides fertile breeding ground for anxiety. While obsessive doubting can exacerbate this anxiety, most obsessive–compulsive defenses help to alleviate the anxiety by shifting attention away from emotion and directing attention to procedure. The resort to rigid, repetitive thought, in particular, often provides therapists, and researchers, a reassuring sense of certainty, even when it is not justified (Nehrig et al. 2019). Obsessive–compulsive defenses are part of our human heritage. They are useful for emotional stability. They contribute to our interest in intellectual understanding, perseverance against obstacles, and the dedication to proper procedure that has advanced our civilization; we should hope and expect to continue to benefit from them. We need them as part of our individual and cultural adaptations, but we will benefit from them more if we can be aware of their presence, and especially of their excesses. Our attention to material reality and time, and our need to understand, order, and categorize, carry the risk of oversimplification and of dispensing with feelings, fantasy, and mind. Rather than using obsessive emphasis on procedure to limit our therapies by overlooking emotion and meaning, we bring emotion and meaning within our scientific purview, broadening our approaches to science and to psychotherapy. This will facilitate our examination of the common elements, mutative elements, and productive and counterproductive elements of different therapies, for different patients. It will orient more of our psychotherapy research to study outcomes that patients care about rather than the usual pre-determined symptom checklists (Shedler and Gnaulati 2020). A very basic additional illustration of the utility a shift from rote procedure to social and emotional attentiveness is found in the observation that female medical doctors listen to their patients more (and stay on schedule less) than their male counterparts (Roter et al. 2002), which may contribute to the lower mortality of their patients (Tsugawa et al. 2017). Likewise, psychotherapies with more listening and less instructing appear to have an advantage (Jones and Pulos 1993).

The mind is a function of the brain, but we all live in our minds. A science of the brain that pays no attention to mind has much more limited use than one that does, but attention to the mind in our current culture is often considered unscientific while the brain seems to hold a scientific endorsement. The recent tendency of people to casually talk about what’s going on in their brains is in fact pseudoscientific: one experiences what occurs in one’s mind, but has no idea what is actually happening in one’s brain. Relatedly, in recent years the field of economics has relinquished the dearly held, if rather suspect, notion of humans as “rational actors” that the field maintained for a century. Even so, this cultural shift in economics is typically called “Behavioral Economics” or “Neuroeconomics” rather than, say, “Psychological Economics,” or “Affective Economics,” i.e., the contribution of mind is alluded to, but not fully acknowledged. A further example of the depreciation of the mind relative to its somatic carrier is that many psychiatrists pay no attention to the biological fact that well-chosen comments affect specific aspects of the mind (and presumably specific circuits in the brain), while focusing more exclusively on medications that disperse to every cell in the body.

Psychiatry and psychology need to take a further step to advance our scientific culture. We need a science of the mind that encompasses the irrational as well as the rational, the unconscious as well as the conscious, and fantasy as well as “reality,” and we need our psychotherapies to follow accordingly. Fantasy, after all, is a major part of our subjective reality, and large part of what motivates us. Keeping this in mind will make for more challenging, interesting, and useful psychiatric and psychological science. This expansion of our science will be much easier to accomplish if we are aware of the ways in which obsessiveness limits our feeling and thinking, thus allowing us to use some of our more adaptive obsessive tendencies to greater advantage. Just as it is difficult to be one’s own therapist, it is difficult to be one’s own anthropologist, but I believe that collective attention to the strengths and weaknesses of our psychiatric and psychological culture will prove a useful endeavor.
